# Supplementary material for: The interactome of intact mitochondria by cross-linking mass spectrometry provides evidence for coexisting respiratory supercomplexes
Source: Mol Cell Proteomics. 2017 Dec 8;17(2):216–32. doi: 10.1074/mcp.RA117.000470 (PMC5795388; doi:10.1074/mcp.RA117.000470)
Supplement: Supplemental Data [file supp_RA117.000470_133922_0_supp_23978_fzffwf.zip › spectra_annotation/mito_spectra_annotation/378-2-9-1-1-1.pdf]

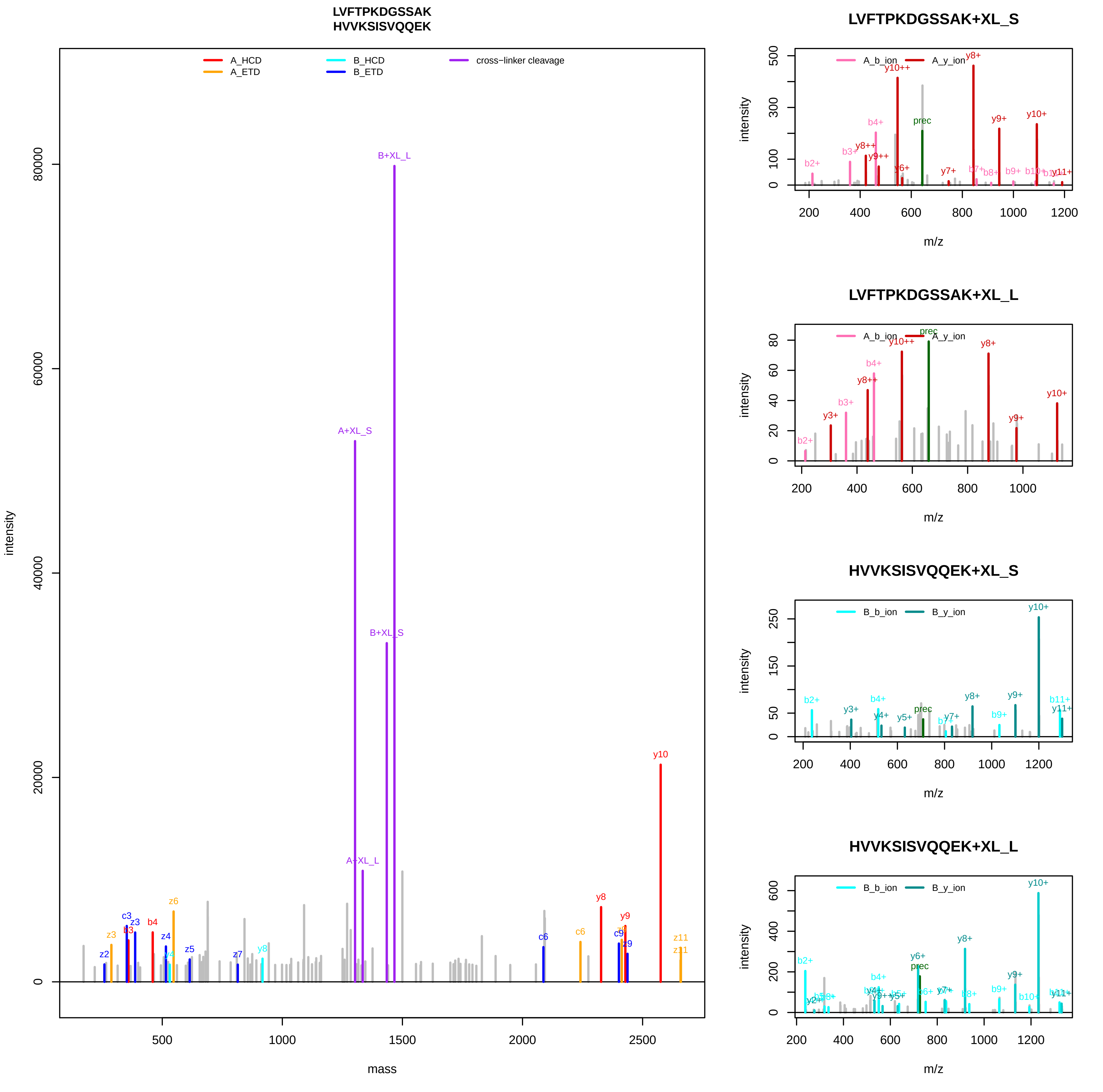
LVFTPKDGSSAK
HVVKSISVQQEK
A_HCD
A_ETD
B_HCD
B_ETD
cross−linker cleavage
B+XL_L
A+XL_S
B+XL_S
A+XL_L
y10
y8
y9
c3
b3
z3
z2
b4
z6
z4
z5
z7
y8
c6
c9
z9
z11
intensity
mass
0
20000
40000
60000
80000
500
1000
1500
2000
2500
LVFTPKDGSSAK+XL_S
A_b_ion
A_y_ion
y8+
y10++
b4+
prec
y9+
y10+
y8++
y9++
b3+
b2+
y6+
y7+
b7+
b8+
b9+
b10+
y11+
intensity
m/z
LVFTPKDGSSAK+XL_L
A_b_ion
A_y_ion
prec
y10++
y8+
b4+
y8++
y10+
b3+
y3+
y9+
b2+
HVVKSISVQQEK+XL_S
B_b_ion
B_y_ion
y10+
y8+
y9+
b2+
b4+
y3+
prec
y4+
y5+
y7+
b9+
b11+
y11+
HVVKSISVQQEK+XL_L
B_b_ion
B_y_ion
y10+
y8+
y6+
prec
b2+
b4+
y9+
b9+
b10+
y11+
intensity
m/z
